# Near-Infrared Emitting Fibers: Stable Jet Electrospinning Flat PbSe Quantum Dots into Poly(methyl methacrylate)

**DOI:** 10.1021/acs.jpclett.5c03441

**Published:** 2026-01-13

**Authors:** Leon Biesterfeld, Dennis Kühn, Fuzhao Li, Franka Gädeke, Dominik A. Rudolph, Frank Schreiber, Peter J. Walla, Ivan Zaluzhnyy, Henning Menzel, Jannika Lauth

**Affiliations:** † Cluster of Excellence PhoenixD (Photonics, Optics, and Engineering−Innovation Across Disciplines), Leibniz University Hannover, D-30167 Hannover, Germany; ‡ Institute of Physical and Theoretical Chemistry, Eberhard Karls University of Tübingen, D-72076 Tübingen, Germany; § Institute of Physical Chemistry and Electrochemistry, Leibniz University Hannover, D-30167 Hannover, Germany; ∥ Institute for Technical Chemistry, 26527 Technische Universität Braunschweig, D-38106 Braunschweig, Germany; ⊥ Institute for Physical and Theoretical Chemistry, Technische Universität Braunschweig, D-38106 Braunschweig, Germany; # Laboratory of Nano and Quantum Engineering, Leibniz University Hannover, D-30167 Hannover, Germany; g Institute of Applied Physics, Eberhard Karls University of Tübingen, D-72076 Tübingen, Germany

## Abstract

Flat colloidal PbSe QDs (fQDs) represent an innovative class of 2D near-infrared (NIR) photoluminescent QDs, which combine extreme thickness with additional lateral confinement. PbSe fQDs exhibit efficient NIR photoluminescence (860–1550 nm) that is adjustable to the low-loss transmission windows of (optical) fibers and makes them highly promising nanoemitters for fiber-based applications. Here, we demonstrate the incorporation of PbSe fQDs into easy-to-handle functional and stable jet electrospun poly­(methyl methacrylate) (PMMA) fibers. Within these electrospun nanocomposites, we find perpendicularly alignedstacks of PbSe fQDs, which give rise to a narrowed and bathochromically shifted photoluminescence (e.g., at 1073 nm, with a quantum yield of 5%) that is caused by an energy transfer into the smallest band gap tail of the PbSe fQD thickness distribution. Embedding PbSe fQDs into solid-state nanocomposite fibers represents an important step forward for implementing near-infrared (NIR)-emitting 2D PbX nanocrystals in fiber optics.

## Graphical abstract

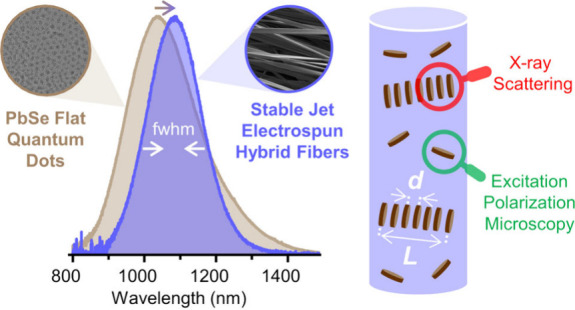

Colloidal semiconductor nanocrystals (NCs) are highly valued for their unique size-tunable properties, while being easy to synthesize, modify and process in solution.^1,2^ In particular, two-dimensional (2D) nanoplatelets (NPLs) and nanosheets with a thickness of a few monolayers (ML) exhibit highly interesting photophysics different from their 0D NC counterparts.^3,4^ For instance, 2D CdSe NPLs^5,6^ have received significant recognition due to their efficient,^7^ color pure^8^ and directed^9^ photoluminescence (PL) throughout the visible range, and consequently have prompted the concurrent exploration of near-infrared (NIR) photoluminescent 2D NCs, such as lead^10–12^ and mercury^13,14^ chalcogenides. Among NIR emitters, 2D lead chalcogenide PbX (X = S, Se, and Te) NPLs^10,15–17^ and flat quantum dots (fQDs)^18^ stand out as a material class with large bulk exciton Bohr radii *a*_B_ (PbS, 20 nm; PbSe, 46 nm; PbTe, ∼82 nm)^19,20^ and interesting properties when forced into the strong confinement regime (*x*,*y*/*a*_B_ < 1 and *z*/*a*_B_ ≪ 1).^21–23^ For example, ultrathin 2D PbS NPLs exhibit linearly polarized, blinking-free, emission (at ∼681 nm) with a significantly narrower line width than their spherical counterparts at low temperatures (down to 0.6 meV with a polarization degree of 0.9).^24^ When using PbSe fQDs rather than CdSe or PbS NPLs, the low-loss transmission windows of optical fibers located further into infrared at ∼850, ∼1350, and ∼1550 nm^25^ can be covered.^15,18,26^ 2D PbSe fQDs exhibit efficient PL at NIR to short-wave infrared wavelengths (860–1550 nm, with up to 61% quantum yield (QY))^26^ and thus have the potential to be used as nanosized light sources in optical fibers, either as classical emitters (e.g., to periodically reamplify a signal that is transmitted through a fiber)^25,27^ or as quantum emitters for emerging quantum information science.^28^ However, colloidal PbSe fQDs (similar to most colloidal NCs in general) face an inherent discrepancy between their finely dispersed colloidal state in solution and the demands of predominantly solid-state applications. Therefore, efficient methods are needed to fabricate macroscopic functional nanocomposites from colloidal NCs. Common strategies for bridging this gap include depositing colloidal solutions of PbX NCs (or NCs in general) in thin films via drop-casting (and doctor-blading),^29^ spin coating,^30^ spray coating,^31^ or dip coating,^30^ and layer-by-layer deposition in conjunction with polymers.^32,33^ Films produced like this have been used as, or have been further processed into, field-effect transistors (ionic-ligand passivated PbSe QDs),^29^ photodetectors (I^–^-capped PbSe NPLs synthesized via cation exchange from CdSe NPLs)^31^ and solar cells (metal halide passivated PbS and PbSe QDs).^30^ More specialized manufacturing options for bringing colloidal NCs into solid-state include, e.g., inkjet printing^34^ or embedding colloidal NCs into electrospun polymer fibers.^35–38^ For the latter, Liu et al. have reported the incorporation of CdSe/ZnS core–shell QDs into disordered electrospun fibers consisting of a polymeric photoresist and have demonstrated their potential as waveguiding structures.^37^ However, an advanced operating mode of electrospinning is stable jet electrospinning (SJES), which allows producing a few micrometers thin, highly aligned unidirectional fibers with macroscopic length scales.^39,40^ With this approach, the chaotic whipping of the spun fiber that is inherent to conventional electrospinning is prevented by adjusting the viscoelastic properties of the spinning solution. In particular, the polymer concentrations and molecular weight must be high enough to achieve the overlap concentration. Typically, the solutions then exhibit non-Newtonian viscoelastic behavior.^41,42^ By adding organic dye molecules^42,43^ or inorganic nanocrystalline emitters^36,43^ to the polymer solution used for SJES, functional composite microfibers can be fabricated via a straightforward yet versatile and cost-efficient approach. The obtained inorganic–organic (or organic–organic) composite fibers are easy to handle and possess the optical functionality of the embedded emitters, which makes them highly interesting for telecommunication applications, waveguiding, and solid-state fiber lasing.^36,42^ We previously embedded green light-emitting (512 nm) colloidal 2D CdSe/CdS core-crown NPLs (4.5 ML) into SJES fibers and found an unexpected perpendicular alignment of the 2D NPLs relative to the fiber. This orientation is beneficial for light harvesting and guiding light along the fiber direction and was found to be caused by normal forces that occur when the viscoelastic spinning solution leaves the spinneret nozzle due to die swell of the polymer.^36,44^ As a consequence, SJES not only represents an advanced method for integrating colloidal NCs into solid-state form but also offers a way to control their orientation by carefully adjusting the rheological properties of the spinning solution via its composition and polymer characteristics, as well as the spinning parameters. However, to the best of our knowledge, nanocomposite (SJES) fibers with similar characteristics containing nanocrystalline emitters with PL at technologically relevant NIR wavelengths, have not yet been demonstrated, despite their potential for (quantum) optical applications.

Here, we report on the incorporation of NIR-emissive 2D PbSe fQDs into poly­(methyl methacrylate) (PMMA) fibers via stable jet electrospinning. The fibers are well-defined with a smooth surface and unidirectional alignment, while exhibiting the narrow and efficient NIR PL of the embedded PbSe fQDs, e.g., at 1073 nm with an fwhm of 185 meV and a PLQY of 5%. Small-angle X-ray scattering reveals that the fQDs are arranged within the fibers such that individual fQDs are stacked in vertical columns, which are then oriented perpendicular to the fiber direction. Contextualizing these findings within a previously derived alignment mechanism driven by the die swell of the polymer advances the understanding of SJES for obtaining solid-state nanocomposites containing macroscopically ordered anisotropic fQDs. The PbSe stack formation results in a bathochromic shift and narrowing of the PL due to energy transfer into the smaller band gap tail of the fQD thickness distribution.

Figures 1a and 1b respectively depict an overview and a high-magnification TEM image of PbSe fQDs, which were synthesized from lead oleate and selenourea by using a method described previously.^15,18,26^ PbSe fQDs used here exhibit average lateral dimensions of (4.6 ± 0.9) nm × (3.5 ± 0.6) nm and a corresponding aspect ratio of 1.3:1, which is in perfect agreement with reported values (see Figure S1a for a lateral size histogram). The higher-magnification micrograph (Figure 1b) reveals the lattice fringes of the individual PbSe fQDs, confirming their highly crystalline nature and cubic rock salt structure (see Figure S1b for a corresponding FFT pattern of a single fQD). The rather low contrast in TEM, compared to conventional spherical QDs, points toward the ultrathin geometry of the fQDs, which has been characterized by scanning tunneling microscopy. We have probed distinct, atomic-layer-defined thicknesses corresponding to monolayered, bilayered, and trilayered PbSe fQDs (see Figure S2 for height/thickness measurements).^18^

**Figure 1:**
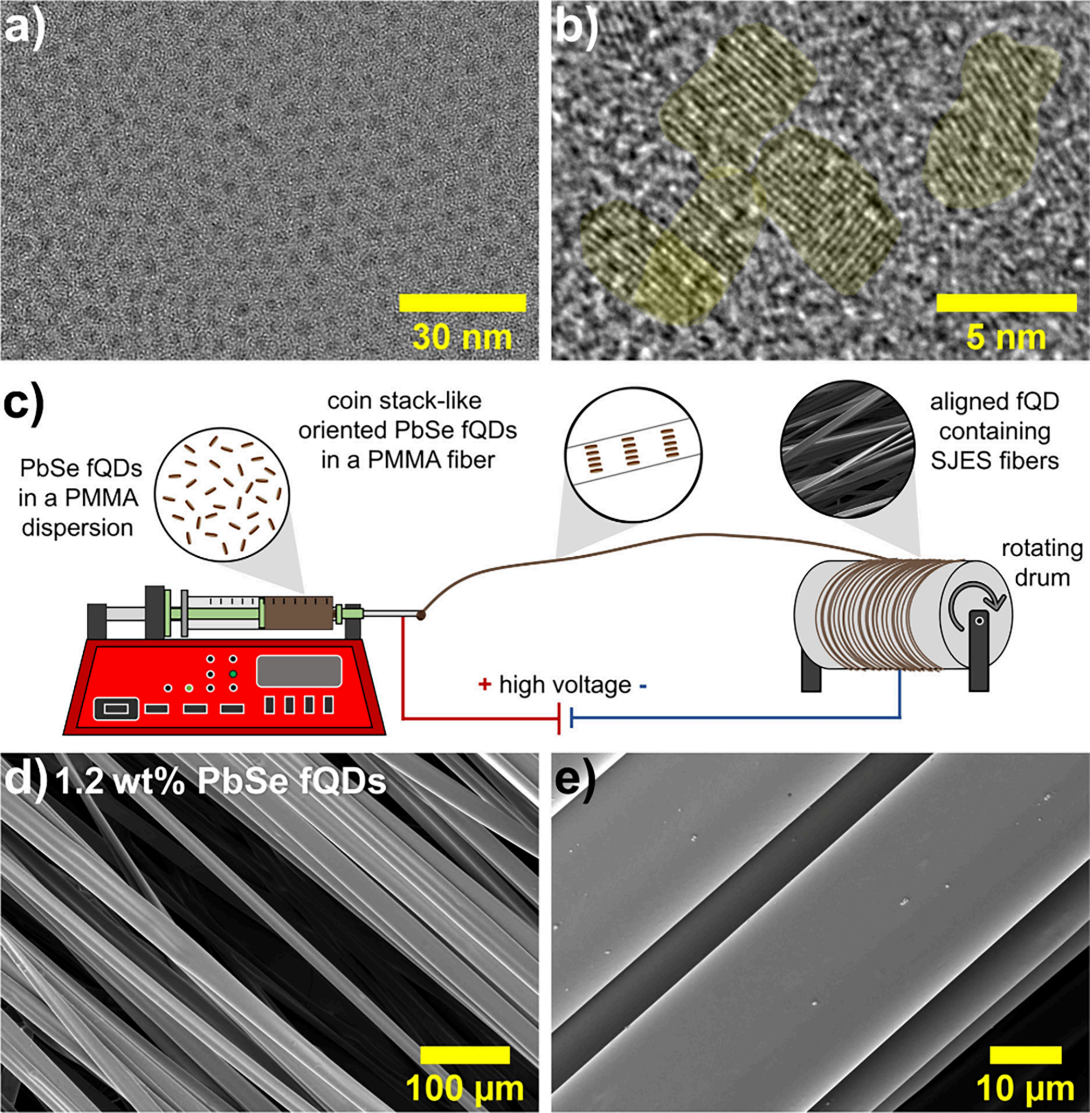
(a, b) TEM images of PbSe fQDs with an average lateral dimension of 4.6 ± 0.9 nm × 3.5 ± 0.6 nm and a slightly rectangular shape (aspect ratio = 1.3:1). (c) Schematic representation of the stable jet electrospinning method used to produce PbSe fQD-containing SJES PMMA fibers. (d, e) SEM images of SJES PMMA fibers containing 1.2 wt % PbSe fQDs. The resulting fibers are highly aligned and evenly shaped, with a smooth surface and an average diameter of 20.3 ± 2.8 μm.

Stable jet electrospinning was performed to embed the PbSe fQDs shown into PMMA fibers (Figure 1c). The spinning solution was prepared by dissolving 35 wt % PMMA in 2-butanone,^36,42^ which was subsequently mixed with 0.6–1.8 wt % PbSe fQDs dispersed in toluene (relative to the PMMA amount). Figure 1c displays a schematic representation of the SJES setup (see Figure S3 for a photograph of the custom-built system). To spin the fibers, an electric field of 30 kV was applied between the steel needle of a syringe, mounted on an automated pump, and a rotating drum collector. The final hybrid microfibers were collected between layers of aluminum foil (see Figure S4 for a photograph). Figures 1d and 1e show SEM images of 1.2 wt % PbSe fQD-containing SJES PMMA fibers. With this PbSe fQD concentration, the parallel aligned fibers show a smooth surface and a rather narrow size distribution, with an average diameter of 20.3 ± 2.8 μm (see Figure S5 for SEM images of fibers containing 0.6 and 1.8 wt % PbSe fQDs). Likewise, the lowest concentration of 0.6 wt % PbSe fQDs results in fibers with a uniform and even surface [*d* = 21.9 ± 3.4 μm], which is essential for minimizing surface-roughness scattering losses when guiding light through the fibers, e.g., in telecommunication applications.^45,46^ These well-defined composite fibers exhibit a ribbon-like cross section (see Figure S6), which is consistent with previous studies on purely organic PMMA SJES fibers without any emitters,^42^ and is presumably related to the drying process of freshly spun fibers.^47,48^ In contrast, adding a high concentration of 1.8 wt % PbSe fQDs to the PMMA spinning solution leads to fibers with a compromised morphology, showing branching between irregularly shaped domains (Figures S5c and S5d). Accordingly, we consider the upper limit of PbSe fQD concentration that can be added to obtain structurally intact SJES fibers to be 1.8 wt % or lower. Notably, the studied concentration range (0.6–1.8 wt %) is considerably higher than all concentrations reported previously by us for SJES fibers containing 2D CdSe/CdS core-crown NPLs (0.003–0.3 wt %), and it highlights not only the versatility of the approach, but also the robustness of stable jet electrospinning for embedding different colloidal NC systems.^36^ In the final fibers, the PMMA matrix fully encapsulates the pristine PbSe fQDs (i.e., without post-synthetic surface passivation), so that the obtained nanocomposite fibers are highly stable under environmental conditions and can be easily handled for further investigation or processing, e.g., by simply cutting out pieces from a fiber mat using scissors. To test the optical functionality of the produced PbSe fQD-containing fibers, we conducted absorption and PL and PL quantum yield measurements on fiber samples and the initial colloidal PbSe fQDs.

Figure 2 shows absorption and PL spectra of colloidal PbSe fQDs (blue) and PbSe fQD-containing SJES fibers (red); their optical characteristics are listed in Table 1. The initial colloidal PbSe fQDs exhibit excitonic absorption at ∼941 nm and PL centered at 1033 nm (Stokes shift of ∼130 meV), with a fwhm of ∼275 meV as well as a PL quantum yield of 18%, which is representative of previous measurements of as-synthesized oleic acid/octylamine passivated PbSe fQDs.^26^ In previous work, we describe that the ensemble PL of PbSe fQDs is governed by three contributions which correspond to the aforementioned monolayer, bilayer, and trilayer PbSe fQD populations.^18^ For the fQDs used here, which were processed into fibers within a few days after the synthesis (i.e., without significant aging), the PL is expected to be dominated almost entirely by bilayer PbSe fQDs (lateral dimensions of ∼4.6 nm × 3.5 nm with a thickness of 1.2 nm), with minor contributions from monolayers and trilayers, causing the ensemble PL to deviate slightly from a symmetric Gaussian shape.

**Figure 2:**
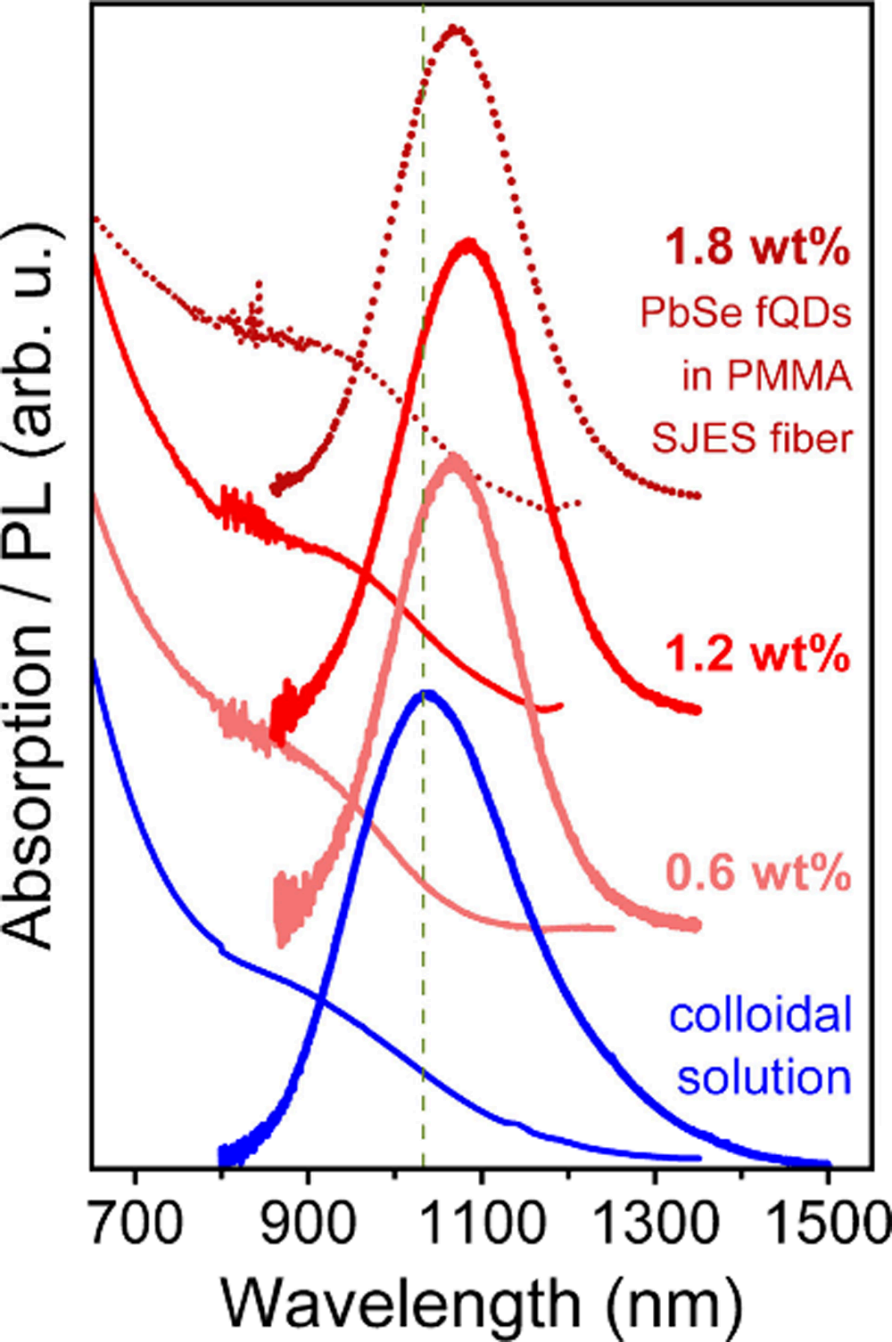
NIR absorption and PL spectra of PbSe fQDs in solution (blue) and in PMMA SJES fibers (red). PbSe fQDs in solution exhibit efficient PL at 1033 nm with a PLQY of 18%, which is retained in the fibers and accompanied by a bathochromic shift to 1070–1085 nm and a PLQY of 3%–6%.

**Table 1:** Excitonic Absorption λ_Abs_ (Based on the Local Minimum of the 2nd Derivative of the Absorption), PL Position λ_PL_, Stokes Shift, fwhm of the PL and PLQY of Colloidal PbSe fQDs and PbSe fQD-Containing PMMA SJES Fibers

sample	λ_Abs_ (nm)	λ_PL_ (nm)	Stokes shift (meV)	PL fwhm (meV)	QY (%)
colloidal PbSe fQDs	941	1033	130	275	18
0.6 wt % fQD fiber	916	1073	198	185	5
1.2 wt % fQD fiber	935	1085	183	190	3
1.8 wt % fQD fiber	939	1070	162	186	6

Regarding the fiber measurements, notably, the optical properties of the 2D PbSe fQDs are retained without any drastic changes for all three nanocomposite samples, which is a prerequisite for any fiber optical application. In detail, the PL of the fibers is bathochromically shifted compared to the colloidal fQDs, with maxima at 1073 and 1085 nm for the 0.6 wt % (light red) and 1.2 wt % PbSe-containing fibers (red), respectively. The PL of the 1.8 wt % fibers (dark red, dotted) is shifted to 1070 nm; however, due to their irregular shapes and rough fiber surfaces, this sample is disregarded in the following (see Figures S5c and S5d). The absorbance features of the fibers are slightly hypsochromically shifted to ∼916 and ∼935 nm with respect to the colloidal fQDs, the fwhm of the fiber PL spectra is decreased by ∼100 meV, and the PLQY is reduced to 5% and 3%, respectively (see Table 1). The decrease in PLQY by a factor of ∼0.22, from 18% to ∼4%, upon embedding the fQDs into the PMMA fiber is consistent with values typically reported for the incorporation of core-only NCs into polymers^33,49^ or their deposition into solid-state thin films,^50,51^ and has been ascribed to changes in the NCs’ surface chemistry exposing previously saturated surface trap states.^52^ This may be overcome in future work by stabilizing the optical properties of PbSe fQDs through additional ligand passivation, for example with a polymer-compatible surface ligand,^52^ and/or by shelling,^53–55^ prior to incorporation into the polymer matrix. The bathochromic PL shift (from 1033 nm to 1073 and 1085 nm) and the thereby increased Stokes shift (from ∼130 meV to 198 and 183 meV), as well as the change to more symmetric Gaussian PL line widths in PbSe fQD-containing fibers, may be attributed to energy transfer between fQDs that are spatially closer to each other and fixed locally in the polymer matrix, compared to the colloidal solution. Voznyy et al. reported on a concentration-dependent energy transfer in colloidal 0D PbS QD ensembles, whereby charge carriers funnel into the lowest-energy tail of the QD distribution, thus increasing the apparent Stokes shift with higher QD concentration.^56^ In the case of the PbSe fQDs studied here, this could be energy transfer from monolayers to bilayers/trilayers and bilayers to trilayers, as well as to laterally larger PbSe fQDs within a given thickness population. Similar energy transfers have been thoroughly studied for coupled QD films,^57,58^ and are amplified by long photoluminescence lifetimes and spatial proximity of (f)­QDs (e.g., due to agglomeration in colloidal solution or stacking in solid state).^56^ For example, Rowland et al. and Guzelturk et al. have demonstrated efficient homofluorescence resonance energy transfer in ordered stacks of 2D CdSe NPLs in which the spatial separation between donor and acceptor is minimized.^59–63^ PbSe fQDs typically exhibit rather long PL lifetimes in the range of 1–2 μs^12,15,26^ and we have previously observed the formation of stacked superlattices with short interparticle distances of ∼0.5 nm for colloidal PbS and PbTe NPLs (chloride/iodide-passivated) when depositing them as thin films.^10,24^ Consequently, energy transfer between PbSe fQDs (stacked or randomly oriented in close proximity) in the fiber is possible and assumed as an additional reason for the lowered PLQY in the fibers, as charge carriers can be transferred to defective fQDs.^60^ It is important to note that this type of energy transfer does not preclude the possibility of the fQDs’ surface chemistry changing simultaneously, thereby causing shifts of their optical features when they are encapsulated in the polymer, as discussed in the context of PLQY.

To gain insight into the relative orientation and the spacing of the PbSe fQDs within the fibers, specifically the possible presence of stacks of PbSe fQDs, we have performed 3D single-particle excitation polarization microscopy (Figure 3) and X-ray diffraction (Figure 4), which are possible due to the comparatively high PbSe fQD concentrations used.

Figure 3a shows polar plots of the radiation patterns of single PbSe fQDs and reference individual 4.5 ML CdSe/CdS core-crown NPLs in SJES PMMA fibers. CdSe NPL-containing SJES fibers were produced as described in ref 36. In brief, the 2D CdSe/CdS NPLs exhibit a lateral size of ∼47 nm × 12 nm × 1.2 nm, meaning that their 2D geometry is much more pronounced than in PbSe fQDs (∼4.6 nm × 3.5 nm × 1.2 nm), which are confined in all three dimensions (with particular strong confinement in the thickness).^18,36^ Regardless of the smaller lateral dimensions in PbSe fQDs, we observe a clear variation in the PL intensity of PbSe fQDs, depending on the polarization excitation light (see Figure 3b), which suggests an anisotropic dipole distribution in PbSe fQDs, different from that of (quasi-)­spherical QDs.^36^ However, this variation is considerably lower than has been observed for CdSe/CdS NPLs, for which the in-plane dipole distribution leads to highly directional PL orthogonal to the NPLs’ plane.^9,36^ Notably, with 3D single-particle excitation polarization microscopy, we only probe individual PbSe fQDs that are not part of a closely packed stack. Therefore, unlike in our previous work on CdSe/CdS NPLs,^36^ it is not possible to draw conclusions about the presence (and possible orientation) of PbSe fQDs inside the SJES PMMA fibers based on optical measurements alone, which is why we have conducted X-ray diffraction experiments.

**Figure 3:**
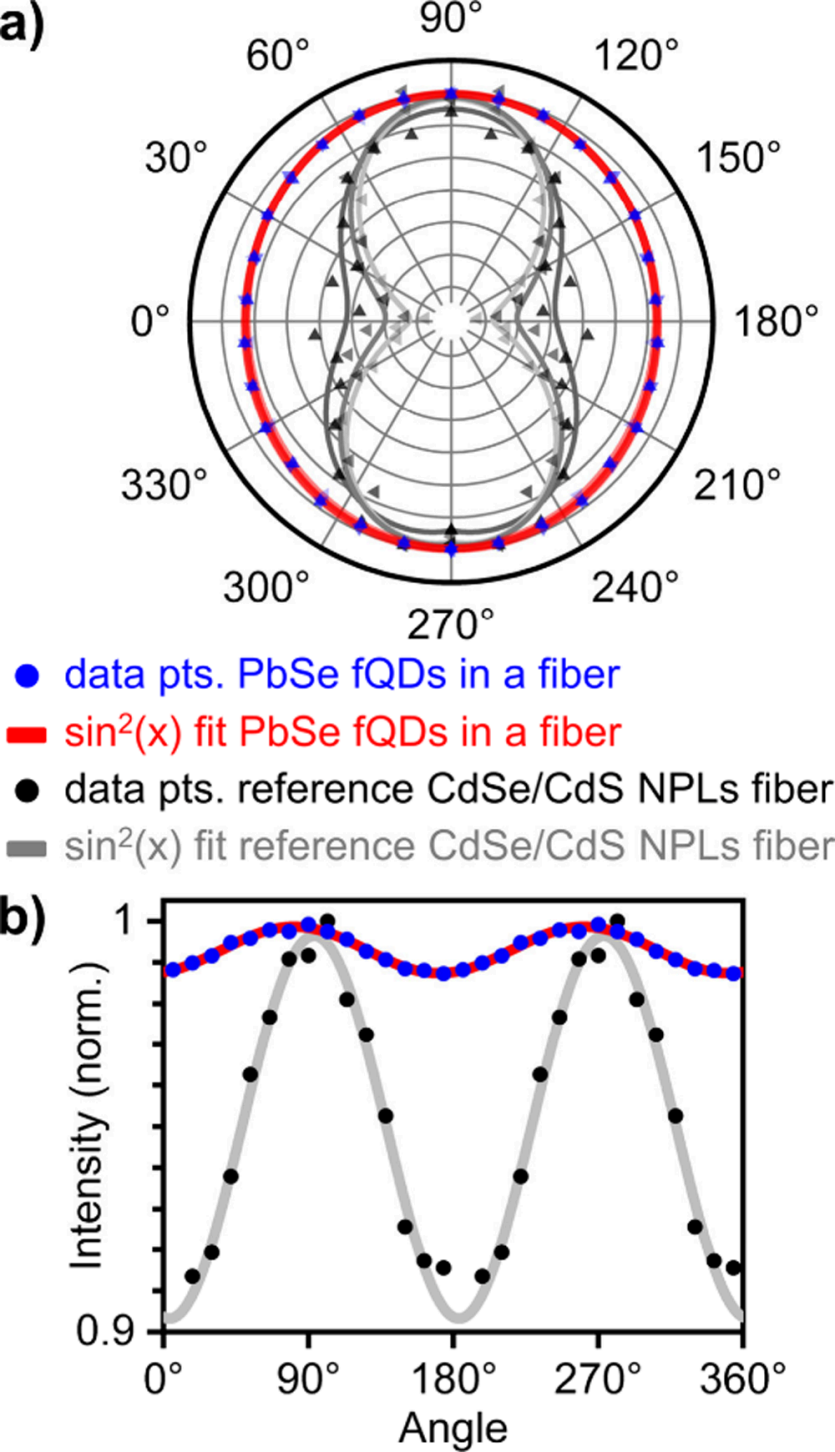
3D single-particle excitation polarization microscopy measurements. (a) Polar plots of the radiation pattern of PbSe fQDs (0.6 wt %) and CdSe NPLs (0.03 wt %) inside a SJES PMMA fiber fitted with a sin^2^(*x*) function (average of 10 analyzed particles). The NPLs/fQDs were excited from three different angles (0°, 120°, and 240°) to ensure excitation of all NPLs/fQDs at the focus point of the objective (shown in three shades of gray for CdSe NPLs; for PbSe fQDs all three red lines overlap). (b) Corresponding averaged sin^2^(*x*) amplitudes.

Figure 4 depicts X-ray diffraction patterns of 0.6–1.8 wt % PbSe fQD-containing SJES PMMA fibers (see Figure S8a for an in-depth description of the 1.8 wt % fiber). All three fiber samples exhibit an anisotropically shaped scattering signal at *q* = $\sqrt{{q_{x}}^{2}+{q_{y}}^{2}}$ ≈ 1.26 nm^–1^, indicating the presence of stacks of PbSe fQDs oriented perpendicular to the fiber axis (see Figures 4a, 4c, and 4e, as well as Figures S9a–S9c; see Figure S10 for X-ray diffraction patterns of complementary drop-casted PbSe-polymer samples containing randomly oriented stacks for comparison). The distance between the PbSe fQDs within a stack can be estimated as *d* = 2π/*q* = 5.0 ± 0.2 nm (Figures 4b, 4d, and 4f, as well as Figure S8b), which is in good agreement with the expected center-to-center distance of stacked 1.2 nm thick fQDs/NPLs separated by oleic acid ligands.^64^ The angular width of the peak at 1.26 nm^–1^ is ∼25° ± 5° for all three mass fractions (see Figures S9d–S9f), indicating a high degree of orientational order of the stacks perpendicular to the PMMA fiber. From a single fQD point of view, this observation differs strikingly from our previous report on the perpendicular orientation of individual CdSe NPLs in SJES PMMA fibers, since each individual PbSe fQD in the present configuration is oriented parallel to the fiber direction.^36^ However, considering the small dimensions of the PbSe fQDs (∼4.6 nm × 3.5 nm × 0.6–1.8 nm) and the dimensions of the formed stacks, it is evident that the same mechanism that vertically aligns CdSe/CdS NPLs can result in a seemingly different arrangement for significantly smaller 2D fQDs: The length of the stack is estimated from the radial width of the scattering peak as *L* = 2π/Δ*q* and decreases from approximately *L* = 65 nm for 0.6 wt % to 40 nm for 1.8 wt %. This means that the rodlike shaped stacks of PbSe fQDs (see Figures 4b, 4d, 4f) exhibit dimensions similar to those of larger individual CdSe/CdS NPLs, and that they show similar behavior when stable jet electrospun with PMMA. We assume that the PbSe fQDs prestack in the spinning solution (a process that is further promoted by the generally high fQD mass fractions used in this work, see Figure S10) with the stacks being randomly oriented within the likewise randomly coiled PMMA chains in solution. At the same time, in the spinneret nozzle the coiled polymer chains become stretched into the flow direction due to the velocity gradient, equally causing the PbSe fQD stacks to orient in the same direction. Upon leaving the nozzle, the polymer chains recoil, inducing a normal force (die swell), that reorients the fQD stacks into an upright orientation perpendicular to the fiber direction.^44^ In this orientation, in turn, each individual PbSe fQD is then oriented parallel to the fiber. For larger PbSe fQD weight fractions, a broad uniform ring emerges in the scattering patterns at *q* ≈ 1.6 nm^–1^ (see Figures 4c and 4e, as well as Figures S8a and S9b,c), corresponding to the appearance of individual PbSe fQDs with no preferred orientation. These findings highlight that the comparatively small single PbSe fQDs cannot be oriented by the polymer and that a higher aspect ratio (as in NPLs) is needed for the normal forces to induce a rotation into an upright position, as is the case for the stacks of PbSe fQDs or individual CdSe/CdS NPLs. These single randomly oriented PbSe fQDs correspond to those observed in 3D single-particle excitation polarization microscopy measurements shown in Figure 3.

**Figure 4:**
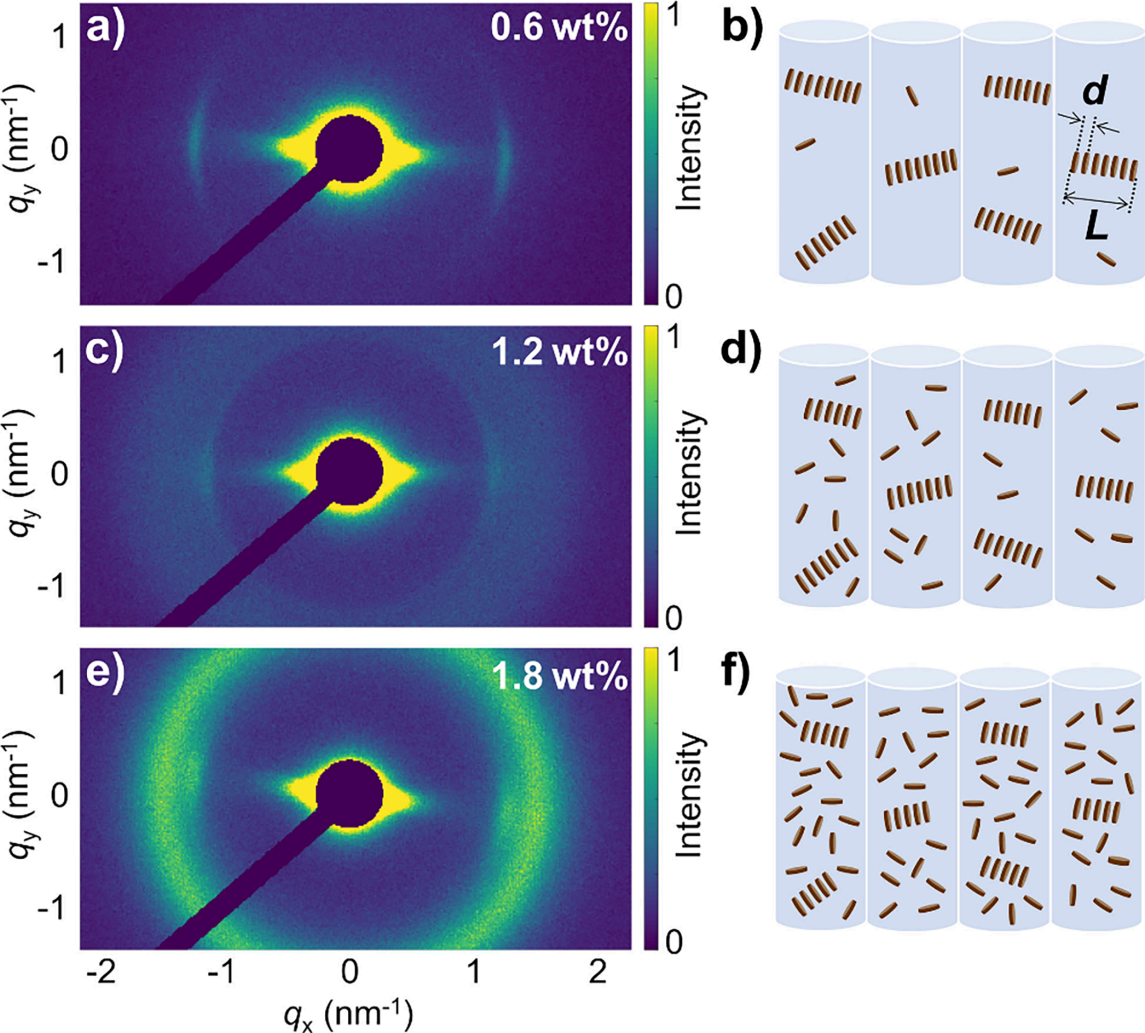
X-ray diffraction experiments conducted on PbSe fQD-containing SJES PMMA fibers. (a, c, e) 2D diffraction patterns of fibers with a PbSe fQD mass fraction of 0.6 wt % (panel (a)), 1.2 wt % (panel (c)), and 1.8 wt % (panel (e)). (b, d, and f) Schematic representation of the arrangement of the PbSe fQDs inside the PMMA fibers.

The stack formation revealed by X-ray scattering underpins the discussed assumption that energy transfer between spatially close PbSe fQDs is the reason for the apparent shift of their PL inside the fibers, compared to that of their initial colloidal state in solution. Since this effect not only shifts the PL to higher wavelengths/lower energies, but is also accompanied by a narrowing of the PL signal, it may be leveraged to tailor the optical properties of the nanocomposite fibers to the second and third telecommunication windows at ∼1350 and ∼1550 nm in the future.

To conclude, we have demonstrated the incorporation of NIR-emissive colloidal 2D PbSe fQDs into stable jet electrospun PMMA fibers. The obtained functional nanocomposite fibers are unidirectionally aligned, have a smooth surface, and possess the optical properties of the embedded PbSe fQDs. For fibers containing 0.6 wt % PbSe fQDs, we measured narrow PL at 1073 nm (fwhm 185 meV) with a quantum yield of 5%, rendering them highly interesting for classical and quantum communication applications. X-ray scattering revealed that the PbSe fQDs in the fibers are distributed in a quasi-hierarchical structure made of stacks of individual fQDs, which are oriented perpendicular to the fiber direction due to die swelling during the SJES process. Within the stacks of PbSe fQDs, the energy transfer toward the smallest band gap tail of the fQD thickness/size distribution results in hybrid fibers with a PL that is narrower than that of their colloidal PbSe fQD building blocks. Our findings underscore the potential of SJES for producing functional hybrid materials and are an important step in developing easy-to-handle solid-state nanocomposites with PL at technologically relevant NIR wavelengths.

## Chemicals

Acetonitrile (≥99.5%), ethanol (EtOH, max. 0.01% H_2_O), *iso*-propanol (≥99.5%), lead­(II) oxide (≥99.99%), methanol (≥99.8%), *n*-octylamine (99%), tetrachloroethylene (TCE, ≥99%), triethylamine (≥99%), trifluoroacetic acid (99%), and trifluoracetic anhydride (≥99%) were purchased from Sigma-Aldrich/Merck. 2-Butanone (>99%) and *n*-hexane (97%) were purchased from Acros Organics. Oleic acid (90%) was purchased from ABCR. Selenourea (99.97%) was purchased from Alfa Aesar. Poly­(methyl methacrylate) (PMMA-8N, 83 000 g/mol) was purchased from Röhm GmbH. *n*-Octylamine and oleic acid were degassed by freeze–pump–thawing prior to being stored and handled inside a N_2_-filled glovebox. All other reagents were used as received from the listed suppliers.

## PbSe Flat Quantum Dot Synthesis

Colloidal PbSe fQDs were synthesized by a method previously described by us.^15,18,26^ The lead oleate used was synthesized via an established protocol by Hendricks et al.^65^ First, a solution of selenourea (193 mg, 1.57 mmol) in octylamine (2.03 mL), oleic acid (0.23 mL), and hexane (0.75 mL) was prepared and stirred at 35 °C for at least 2 days prior to the fQD synthesis. To synthesize the PbSe fQDs, lead oleate (1.83 mg, 2.7 mmol) was dissolved in a mixture of octylamine (2 mL), oleic acid (4 mL), and hexane (18 mL) at 35 °C. After complete dissolution (∼5 min), the sealed mixture was cooled to 0 °C using an ice bath. The selenourea solution (2.5 mL) was then quickly injected into the vigorously stirred lead oleate mixture. After 10 min of reaction time, the dark brown reaction mixture was quenched by the addition of dry EtOH (18.5 mL). To purify the already destabilized PbSe fQDs, dry EtOH was added dropwise, until a precipitate formed at the bottom of the flask. This mixture was then centrifuged at 2500 rcf, the supernatant was discarded, and the precipitate was redispersed in dry toluene (10 mL). This process was repeated two more times before sealing and storing the purified PbSe fQDs under a N_2_ atmosphere. All steps of the PbSe fQD synthesis, except for cooling, were performed under inert gas conditions in a N_2_-filled glovebox.

## Stable Jet Electrospinning of PbSe fQD-Containing PMMA Fibers

SJES was performed using a custom-built electrospinning setup (see Figure S3 for a photograph of the setup and details about its components). The spinning solution was prepared by stirring 35 wt % of PMMA in 2-butanone for 24 h at room temperature, resulting in a clear, viscous solution. Then, PbSe flat quantum dots dispersed in toluene were added at varying concentrations (0.6–1.8 wt % of Pb) and ultrasonicated for 10 min prior to spinning. For SJES, 4 mL of the PMMA/fQD dispersion was loaded into a syringe equipped with a stainless-steel needle (inner diameter of 0.8 mm) and mounted on an automated syringe pump. The rotating drum collector, which was covered with aluminum foil to make fiber removal easier, was placed 30 cm from the needle tip inside the SJES chamber. Prior to spinning, nitrogen was used to flush the chamber atmosphere to maintain a relative humidity of approximately 10%. A +15 kV voltage was applied to the steel needle and a voltage of −15 kV was applied to the drum collector, resulting in a total potential difference of 30 kV across the spinning gap. The solution flow rate was kept at 25 mL/h, and the drum collector was rotated at 2000 rpm to improve fiber alignment. All parameters were kept constant across the experiments. After each spinning run, the chamber was flushed with nitrogen for 15 min to remove any residual organic solvent vapors.

## Transmission Electron Microscopy

TEM images were obtained using an FEI Tecnai G2 F20 transmission electron microscope with a field emission gun operating at 200 kV. For this, the colloidal PbSe fQDs were crop-cast onto carbon-coated copper grids (300 mesh) from Quantifoil. The average lateral size of the PbSe fQDs was determined using the ImageJ software.

## Scanning Electron Microscopy

To image the fiber surfaces, a 1 cm × 1 cm section was cut from the electrospun fiber mat and mounted on an aluminum SEM sample carrier by using conductive adhesive carbon tape. To obtain cross-sectional images, another 1 cm × 1 cm fiber sample was placed between two layers of carbon tape and immersed in liquid nitrogen for 1 min. The frozen sample was then broken at the midpoint between the tapes to expose the cross-section, after which it was mounted on a sample carrier. To improve the conductivity, all samples were sputter-coated with a thin layer of gold using a Leica Model SCD 050 sputter coater (from Leica Microsystems). SEM images were captured using a combined system consisting of a Zeiss Model EVO LS 25 and Bruker Model EVO system at magnifications ranging from 500× to 35 000×. The average diameter of the electrospun fibers was determined using the ImageJ software. For each sample, SEM images acquired at different locations and orientations were measured using manual line profiling across well-resolved fibers (sample size *n* = 40) and the values were then averaged to obtain the mean fiber diameter.

## NIR Photoluminescence and UV-vis-NIR Absorption Spectroscopy

Near-infrared photoluminescence spectra were acquired by using an Edinburgh FLS 1000 UV–vis-NIR spectrometer equipped with a 450 W ozone-free xenon arc lamp for excitation. The PL was monitored using an InGaAs NIR photomultiplier tube (Model 1650 detector cooled with liquid N_2_) from Edinburgh Instruments. Colloidal samples for optical ensemble spectroscopy were prepared by diluting the colloidal PbSe fQD solutions in TCE (with an optical density below 0.2 at 500 nm) in a quartz cuvette (quartz glass, high-performance Model QS 200 – 2500 nm, with an optical path length of 1 cm by Hellma). For fiber samples, a 3 cm × 1 cm sample was cut from the electrospun fiber mat and placed on the solid sample holder of the FLS 1000 system. Absolute PLQYs were determined using an integrating sphere with the FLS 1000 system. For this, the scattering at 450 nm and the PL in the NIR of TCE or a blank PMMA fiber sample and the fQDs or PMMA/fQD fibers, respectively, were measured separately, accounting for the difference in the sensitivity of both detectors with a correction factor. UV-vis-NIR absorption spectra were collected using a double beam Cary 5000 spectrophotometer from Agilent Technologies that was equipped with a tungsten halogen (Vis) lamp and a deuterium arc (UV) lamp and a PbSmart NIR detector for monitoring. For fiber samples, an integrating sphere (diffuse reflectance accessory from Agilent Technologies) was used. The absorption maxima/shoulders were determined by the local minima of the second derivative of the smoothed absorption (locally estimated scatterplot smoothing). The increased noise present in all absorption spectra around 800 nm stems from the NIR detector changes at this wavelength.

## Excitation Polarization Microscopy

For 3D excitation polarization microscopy, PbSe fQD-(0.6 wt %) and CdSe NPLs-containing (0.03 wt %) PMMA SJES fibers were placed between a microscope slide and a coverslip and fixated using Norland Optical Adhesive 148 glue (Norland Products). The NCs were excited using a Coherent Chameleon Ultra II laser that generates 900 nm light, which was frequency-doubled to 450 nm by using a β-BaB_2_O_4_ crystal (Thorlabs). The beam was broadened using a lens system and the polarization of the laser beam was rotated using a rotating half-wave plate (achromatic λ/2 plate, 400–800 nm, Thorlabs). Sending the beam through a rotatable wedge prism (4° beam deviation, 375–700 nm, Thorlabs), allowed entering the back aperture of the objective (NA = 1.35, oil immersion, UPlanApo, 60×, Olympus) from three different positions, thus hitting the sample from three different angles. The emitted light passed through a dichroic mirror (SP 556, AHF) and onto an electron-multiplying charge-coupled device camera (iXonEM + 897, back-illuminated, Andor Technology). Consequently, the intensity of the radiation pattern is a function of the excitation angle. It is important to note that this method only analyzes individual fQDs: stacks are not taken into account.

## Small-Angle X-ray Scattering

X-ray diffraction experiments were performed by using a Xeuss 2.0 instrument with a copper anode. The diffraction patterns from the vertically aligned fibers were recorded by a Pilatus 300 K detector in a transmission geometry. The X-ray beam was focused to approximately 1.2 nm × 1.2 nm. The direct beam was covered with a beam stop.

## Supplementary Material



## Notes

Lateral size histogram of PbSe fQDs, high-magnification TEM image and FFT pattern of a PbSe fQD; scanning tunneling microscopy thickness measurements of PbSe fQDs; photograph of the SJES setup; photograph of PbSe fQD-containing SJES PMMA fibers; additional SEM overview images of SJES PMMA fibers containing 0.6 and 1.8 wt % PbSe fQDs; cross-sectional SEM images of 0.6-and 1.2-wt %-containing fibers; excitation polarization microscopy images of the 0.6 wt %-containing fibers; SAXS diffraction pattern of 1.8 wt %-containing fibers, *d* vs wt % plot, and ΔΦ vs wt % plot; *I*_rad_ vs *q* plots and *I*_ang_ vs Φ plots for all three fiber samples; X-ray diffraction patterns of complementary drop-casted PbSe fQD-polymer samples (PDF)

## ABBREVIATIONS

2D: two-dimensional
Abs: absorption
EtOH: ethanol
FFT: fast Fourier transform
fQD: flat quantum dot
fwhm: full width at half-maximum
ML: monolayer
NA: numerical aperture
NC: nanocrystal
NIR: near-infrared
NPL: nanoplatelet
PL: photoluminescence
PMMA: poly­(methyl methacrylate)
QY: quantum yield
rcf: relative centrifugal force
SAXS: small-angle X-ray scattering
SEM: scanning electron microscopy
SJES: stable jet electrospinning
TCE: tetrachloroethylene
TEM: transmission electron microscopy
UV: ultraviolet
Vis: visible
