# Supplementary material for: Near-Infrared Emitting Fibers: Stable Jet Electrospinning Flat PbSe Quantum Dots into Poly(methyl methacrylate)
Source: J Phys Chem Lett. 2026 Jan 13;17(4):955–65. doi: 10.1021/acs.jpclett.5c03441 (PMC12862810; doi:10.1021/acs.jpclett.5c03441)
Supplement: Supplementary file 1 [file jz5c03441_si_001.pdf]

Supporting Information

Near-Infrared Emitting Fibers: Stable Jet Electrospinning Flat PbSe Quantum Dots with Poly(methyl methacrylate)

Leon Biesterfeld,[a,b,c] *Dennis Kühn,*[d] *Fuzhao Li,*[a,d] *Franka Gädeke,*[e] *Dominik A. Rudolph,*[a,c,f]

Frank Schreiber,[g] *Peter J. Walla,*[a,e] *Ivan Zaluzhnyy,*[g] *Henning Menzel,*[a,d] *Jannika Lauth* *[a,b,c,f]

a – Cluster of Excellence PhoenixD (Photonics, Optics, and Engineering – Innovation Across Disciplines), D-30167 Hannover, Germany.

b – Institute of Physical and Theoretical Chemistry, Eberhard Karls University of Tübingen, D-72076 Tübingen, Germany.

c – Institute of Physical Chemistry and Electrochemistry, Leibniz University Hannover, D-30167 Hannover, Germany.

d – Institute for Technical Chemistry, Technische Universität Braunschweig, D-38106 Braunschweig, Germany.

e – Institute for Physical and Theoretical Chemistry, Technische Universität Braunschweig, D-38106 Braunschweig, Germany.

f – Laboratory of Nano and Quantum Engineering, Leibniz University Hannover, D-30167 Hannover, Germany.

g – Institute of Applied Physics, Eberhard Karls University of Tübingen, D-72076 Tübingen, Germany.

*jannika.lauth@uni-tuebingen.de

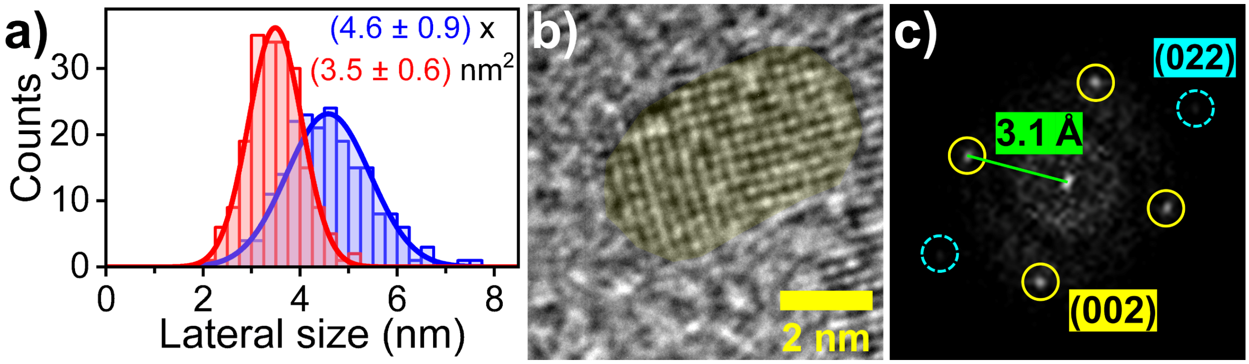

Figure S1. (a) Lateral size histogram of the PbSe fQDs used in this study (sample size $n = 200$). The x-lengths (blue) correspond to the longest dimension of the fQDs and the y-widths (red) were determined by measuring the longest perpendicular distance. (b) High-magnification TEM image of a single PbSe fQD. (c) FFT pattern of the PbSe fQD shown in (b), composed of a set of diffraction peaks that are characteristic of cubic rock-salt-structured PbSe (with a lattice constant $a = 6.1$ Å and space group $Fm\bar{3}m$, as referenced in PDF card 01-077-0245).

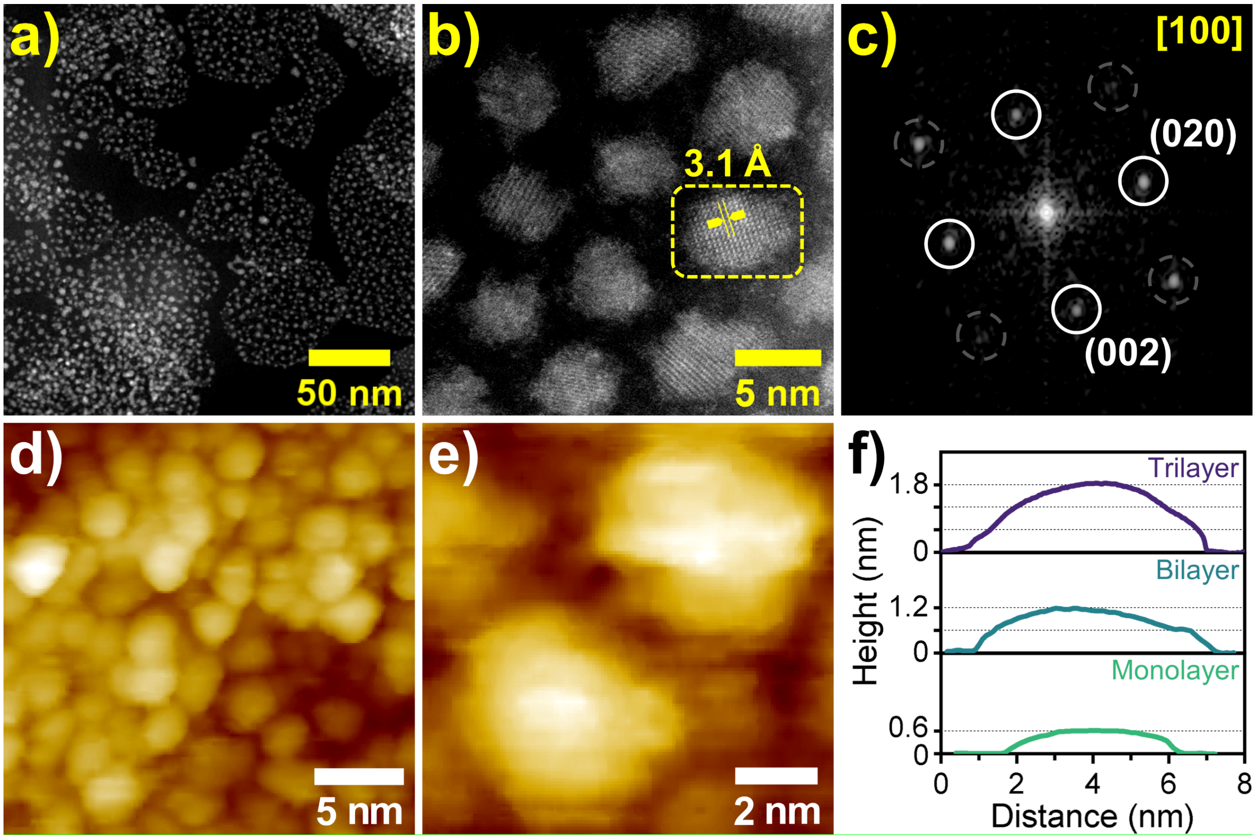

Figure S2. Overview on the morphology of PbSe fQDs, as previously published.[1] (a, b) HR-HAADF-STEM images of PbSe fQDs. (c) FFT of the region marked in (b). (d, e) STM images (V_S = 4 V; I_{set} = 50 pA) of PbSe fQDs (shown in (a) and (b)). **(f) STM height profiles of colloidal PbSe fQDs showing distinct atomic layer-defined thicknesses of 1.8, 1.2 and 0.6 nm, corresponding to tri- to monolayer cubic PbSe, respectively.**

Reprinted with permission from *Nano Lett.* **2025**, *25* (31), 12019–12024. Copyright © 2025 American Chemical Society. Further permissions related to the material excerpted should be directed to the American Chemical Society.

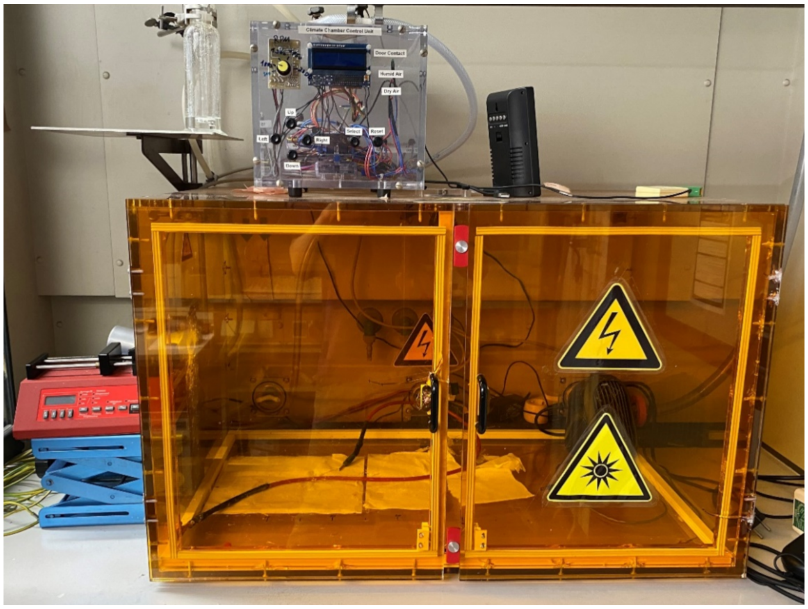

Figure S3. Custom-built stable jet electrospinning setup used to fabricate PbSe fQD-containing PMMA SJES fibers, consisting of: a tinted acrylic glass chamber (80 x 50 x 50 cm^3), two high-voltage generators (from Heinzinger electronic) to apply an electric potential, a spinneret (21-gauge needle from B. Braun) connected to the positive voltage, and a custom-made rotating drum collector (hollow aluminum cylinder with 10 cm diameter and 17 cm length, rotated at 8–33 Hz) connected to the negative voltage. The spinning solution was injected *via* a syringe pump LA-30 (left side) from Landgraf Laborsysteme HLL.[2] The gas atmosphere inside the orange chamber was controlled using a custom-built Arduino-based climate chamber control unit (top).

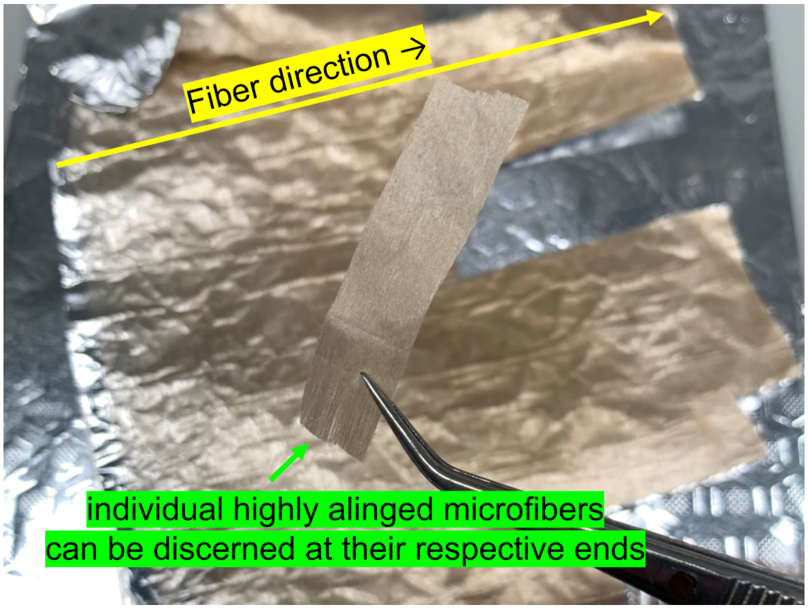

Figure S4. Photograph of stable jet electro spun PbSe fQD-containing PMMA microfibers on aluminum foil (used to facilitate fiber separation).

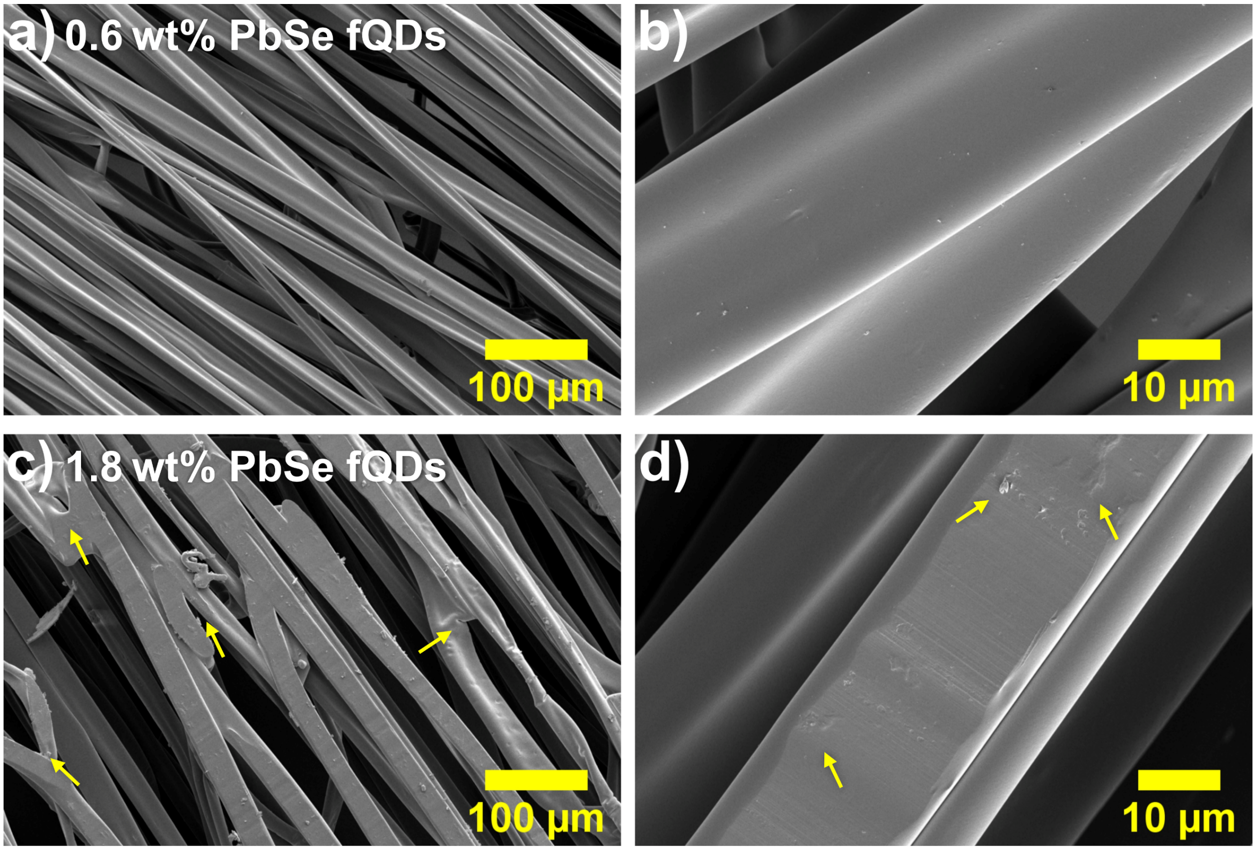

Figure S5. SEM overview images of SJES PMMA fibers containing 0.6 wt% (a, b) and 1.8 wt% PbSe fQDs (c, d). At the lower PbSe fQD concentration, the aligned fibers exhibit a smooth, regular surface and a mean diameter of (21.9 ± 3.4) µm, which is similar to the 1.2 wt% sample shown in Figure 1d,e of the main manuscript. However, adding 1.8 wt% PbSe fQDs results in unevenly shaped SJES fibers with a rougher surface, as well as considerable crosslinking between adjacent fibers and splitting of individual fibers (indicated by the yellow arrows in panel c). Consequently, 1.8 wt% PbSe fQDs are considered the upper limit for obtaining high-quality PbSe fQD-containing SJES PMMA fibers within the parameter space of this work.

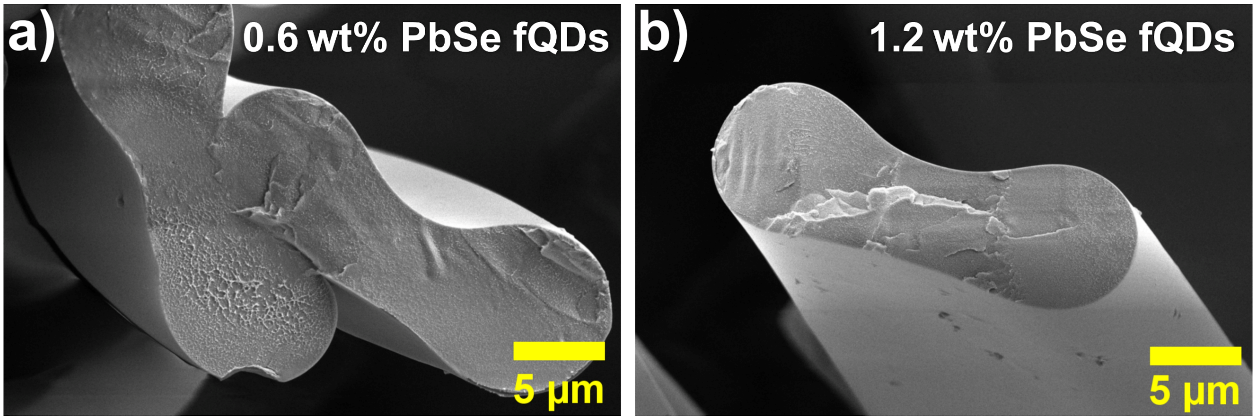

Figure S6. Cross sectional SEM images of individual PbSe fQD-containing fibers highlighting their ribbon-/dog-bone-like shape.

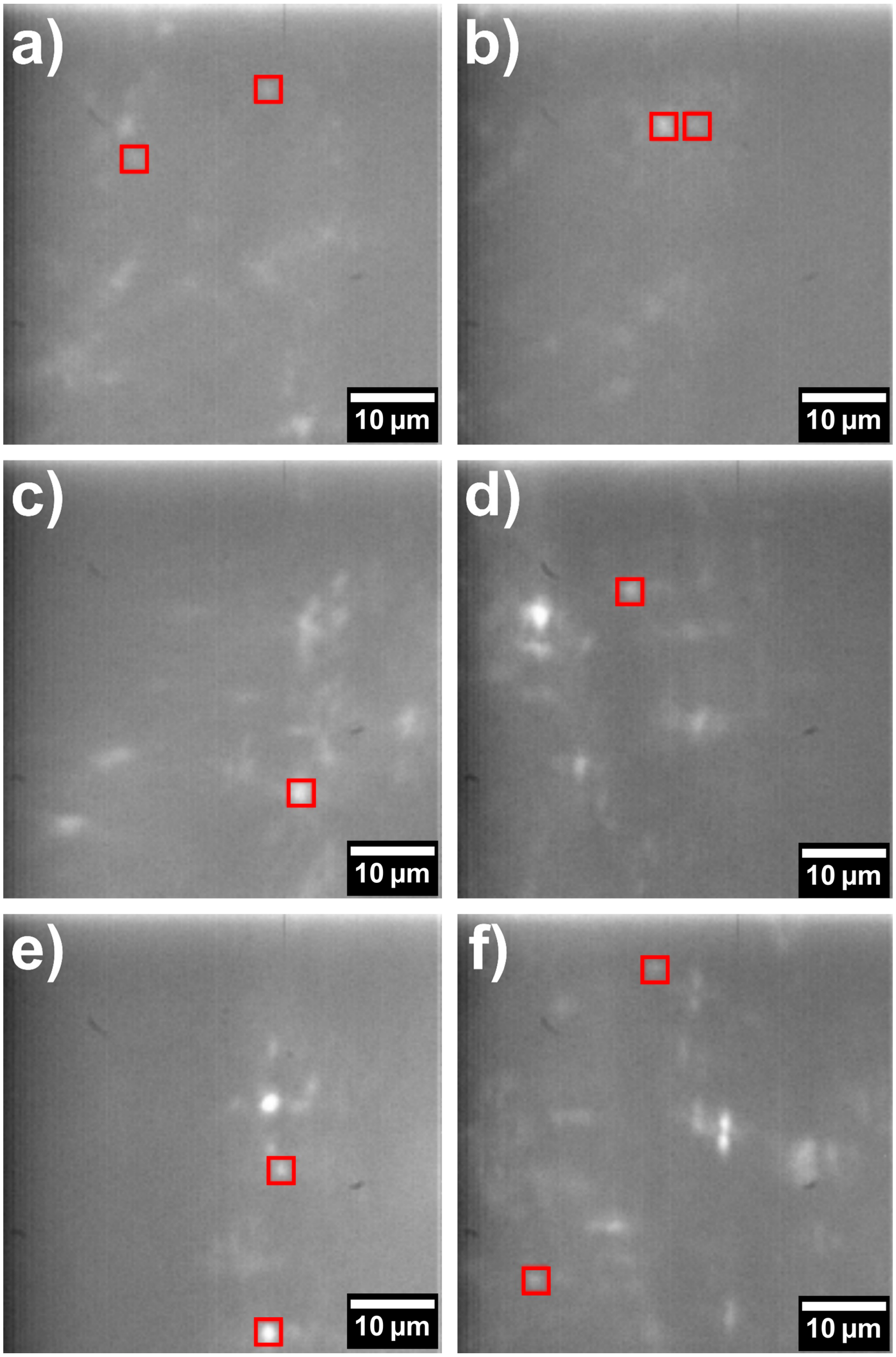

Figure S7. 3D single-particle excitation polarization microscopy images. The data shown in Figure 3 of the main manuscript were collected by analyzing the signal from the spots indicated in red. Spots with a regular round shape and clear sinusoidal modulation of the signal when varying

the polarization orientation were selected for analysis. Spot brightness does not play a role in the selection process, because the brightness only reflects the angle-dependent absorption probability of a given fQD; therefore, different excitation angles lead to different brightness levels.

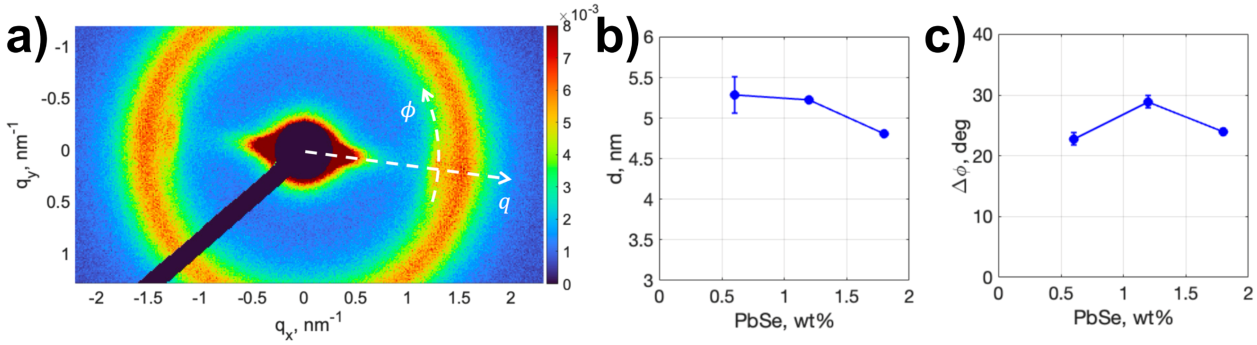

Figure S8. (a) SAXS diffraction pattern of SJES fibers containing 1.8 wt% PbSe fQDs. The white dashed lines indicate the radial (q) and angular (ϕ) directions in polar coordinates. The scattering signal of PbSe fQDs within stacks is visible at $q = 1.3$ nm^{-1}. This signal can be used to estimate the distance between the PbSe fQDs in a stack and their orientational order. The uniform scattering ring at $q = 1.5$ nm^{-1} originates from the unordered individual PbSe fQDs. This ring is only visible for the samples with PbSe fQD mass fractions of 1.2 and 1.8 wt%. (b) Distance d between the PbSe fQDs within a stack, determined from the radial position of the scattering peak, plotted against the PbSe fQD weight fractions. (c) Fwhm in angular direction of the scattering peak originating from the PbSe stack, plotted against the PbSe fQD weight fractions.

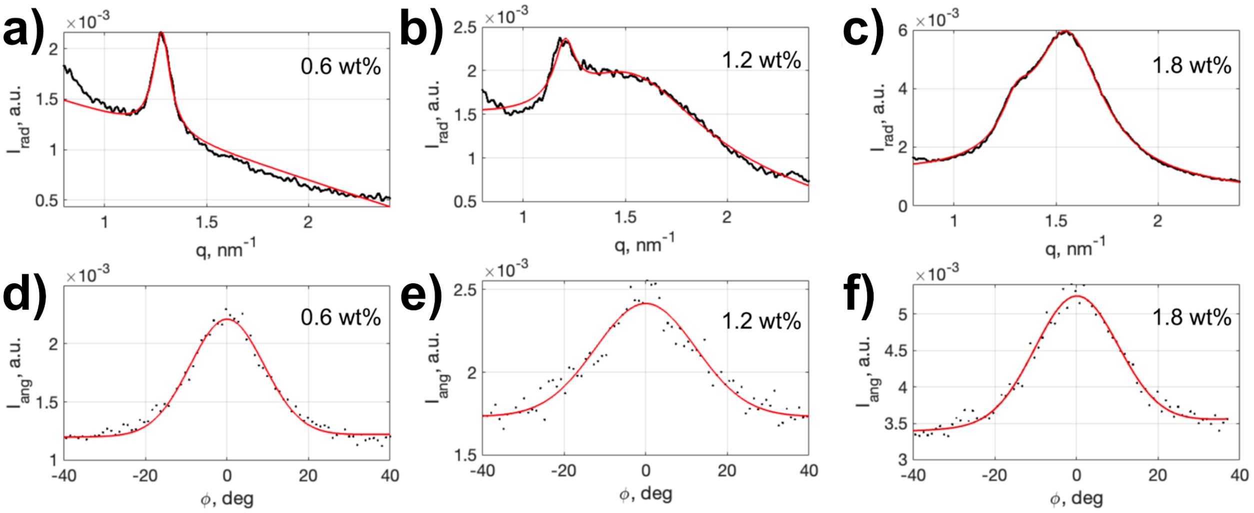

Figure S9. Radial (a–c) and angular (d–f) cross-sections of the scattered intensity through the peak corresponding to the stacks of PbSe fQDs in fibers with PbSe fQD mass fractions of 0.6 wt% (a, d), 1.2 wt% (b, e) and 1.8 wt% (c, f). Black points represent the experimental data, and the red lines correspond to fits with Gaussian functions with a linear background. For the radial profiles at 1.2 wt% and 1.8 wt% (panels (b) and (c)), two Gaussian functions were used to take into account two contributions: (1) PbSe fQDs within a stack (small peak at around $q = 1.3$ nm^{-1}) and (2) individual PbSe fQDs (large peak at around $q = 1.5$ nm^{-1}). The results of the fits, *i.e.* the corresponding distances between the PbSe fQDs and the angular fwhm of the peaks, are shown in Figure S7b, c above.

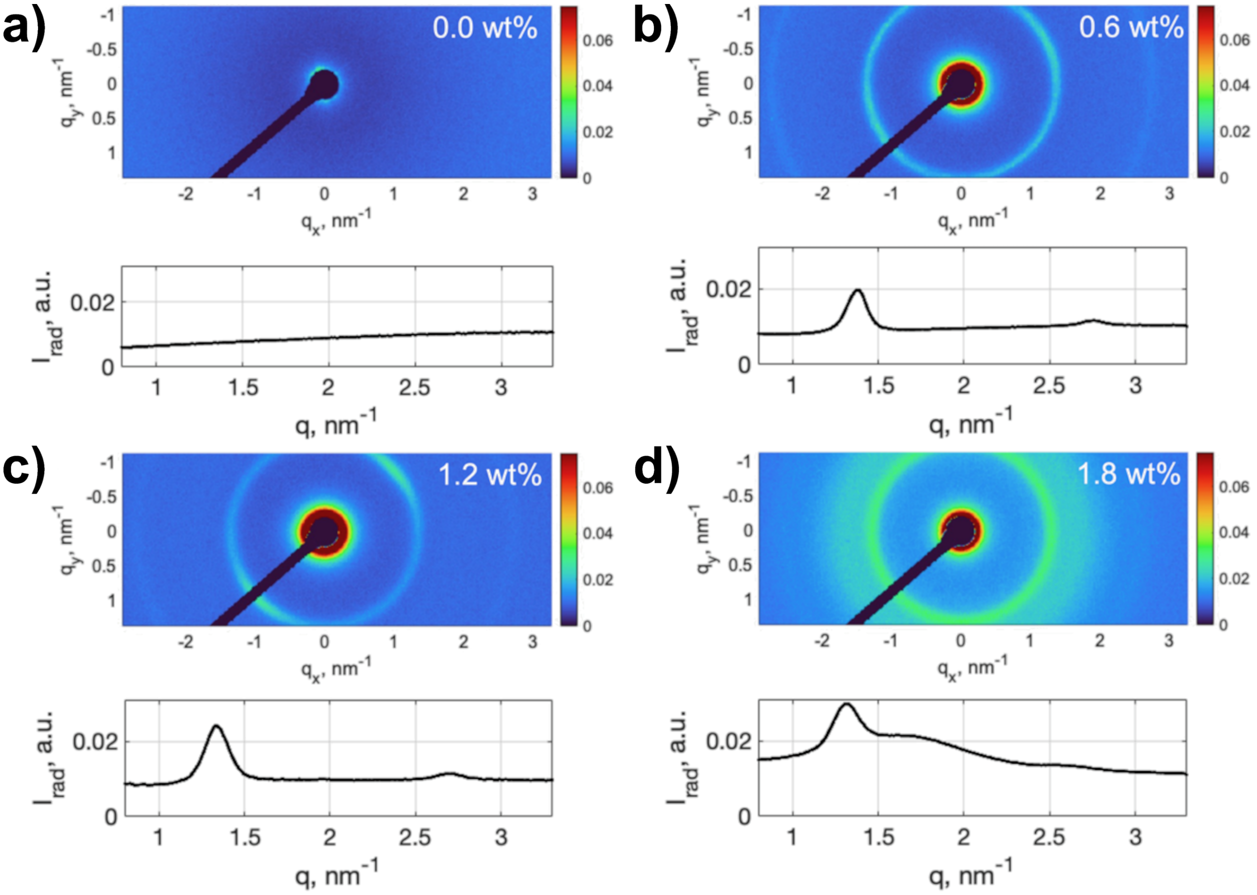

Figure S10. X-ray diffraction patterns of complementary drop-casted chip-like PbSe fQD-polymer samples of the same composition that was used for the SJES fibers. All samples containing PbSe fQDs (b–d) exhibit an isotropic scattering ring at $q \approx 1.34$ nm$^{-1,}$ corresponding to stacks of PbSe fQDs with a random orientation (indicated by an isotropic ring instead of an anisotropically shaped scattering signal). The interparticle spacing within these stacks is similar to that observed in the SJES fibers (~4.7 nm here *vs* ~5.0 nm in the fibers), supporting the assumption that the PbSe fQDs pre-stack in the spinning solution, while the alignment of the stacks is an effect of the SJES technique. (The minor anisotropy of the scattering signal in panel (c) is presumably caused by accidental stress during sample preparation, because the spinning solution is optimized for SJES rather than for drop-casting and drying under ambient conditions.)

REFERENCES

(1) Biesterfeld, L.; Ngo, H. T.; Addad, A.; Rudolph, D. A.; Leis, W.; Seitz, M.; Ji, G.; Grandidier, B.; Delerue, C.; Lauth, J.; Biadala, L. Monolayer-Defined Flat Colloidal PbSe Quantum Dots in Extreme Confinement. *Nano Lett.* **2025**, *25* (31), 12019–12024.

(2) Christ, H.-A.; Ang, P. Y.; Li, F.; Johannes, H.-H.; Kowalsky, W.; Menzel, H. Production of Highly Aligned Microfiber Bundles from Polymethyl Methacrylate via Stable Jet Electrospinning for Organic Solid-State Lasers. *J. Polym. Sci.* **2022**, *60* (4), 715–725.
